# RETRACTION: A Severe Alzheimer’s Disease Patient Improved by Intravenous Mesenchymal Stem Cell Transplant

**DOI:** 10.1155/crnm/9873498

**Published:** 2026-07-16

**Authors:** Case Reports in Neurological Medicine

**RETRACTION**: T. H. Pazili, “A Severe Alzheimer’s Disease Patient Improved by Intravenous Mesenchymal Stem Cell Transplant,” *Case Reports in Neurological Medicine* 2024, no. 1 (2024): 1–5, https://doi.org/10.1155/2024/8353492.

The above article, published online on 14 July 2024 in Wiley Online Library (https://wileyonlinelibrary.com), has been retracted by agreement between the author; the handling Editor, Isabella Simone, and John Wiley & Sons Ltd. The retraction has been agreed due to the below concerns being raised by the author:

• The patient described had diabetes, not Alzheimers. The author claims this was a mistranslation; however, the symptoms described in the case report are of a patient with Alzheimers.
• The author did not get consent from the laboratory for publishing this case.
• The case description is not correct, as the patient did not live in a nursing home.

The author was not able to confirm that written informed consent obtained had been obtained from the patient for the publication of this case report, and the article has therefore been removed given this uncertainty.

The author did not respond when asked for further clarification, and after assessment by the editorial board the article is retracted.

